# NMY-2, TOE-2 and PIG-1 regulate *Caenorhabditis elegans* asymmetric cell divisions

**DOI:** 10.1371/journal.pone.0304064

**Published:** 2024-05-24

**Authors:** Joseph Robinson, Jerome Teuliere, Shinja Yoo, Gian Garriga

**Affiliations:** Department of Molecular and Cell Biology, University of California Berkeley, Berkeley, CA, United States of America; University of Kansas College of Liberal Arts and Sciences, UNITED STATES

## Abstract

Asymmetric cell division is an important mechanism that generates cellular diversity during development. Not only do asymmetric cell divisions produce daughter cells of different fates, but many can also produce daughters of different sizes, which we refer to as Daughter Cell Size Asymmetry (DCSA). In *Caenorhabditis elegans*, apoptotic cells are frequently produced by asymmetric divisions that exhibit DCSA, where the smaller daughter dies. We focus here on the divisions of the Q.a and Q.p neuroblasts, which produce larger surviving cells and smaller apoptotic cells and divide with opposite polarity using both distinct and overlapping mechanisms. Several proteins regulate DCSA in these divisions. Previous studies showed that the PIG-1/MELK and TOE-2 proteins regulate DCSA in both the Q.a and Q.p divisions, and the non-muscle myosin NMY-2 regulates DCSA in the Q.a division but not the Q.p division. In this study, we examined endogenously tagged NMY-2, TOE-2, and PIG-1 reporters and characterized their distribution at the cortex during the Q.a and Q.p divisions. In both divisions, TOE-2 localized toward the side of the dividing cell that produced the smaller daughter, whereas PIG-1 localized toward the side that produced the larger daughter. As previously reported, NMY-2 localized to the side of Q.a that produced the smaller daughter and did not localize asymmetrically in Q.p. We used temperature-sensitive *nmy-2* mutants to determine the role of *nmy-2* in these divisions and were surprised to find that these mutants only displayed DCSA defects in the Q.p division. We generated double mutant combinations between the *nmy-2* mutations and mutations in *toe-2* and *pig-1*. Because previous studies indicate that DCSA defects result in the transformation of cells fated to die into their sister cells, the finding that the *nmy-2* mutations did not significantly alter the Q.a and Q.p DCSA defects of *toe-2* and *pig-1* mutants but did alter the number of daughter cells produced by Q.a and Q.p suggests that *nmy-2* plays a role in specifying the fates of the Q.a and Q.p that is independent of its role in DCSA.

## Introduction

A core aspect of development is that a single cell can give rise to multiple cell types. This is often accomplished by Asymmetric Cell Division (ACD), where a cell divides to produce daughters with distinct fates [1]. One mechanism contributing to ACD is the asymmetric distribution of cell fate determinants that specify daughter cell fates. Another mechanism contributing to ACD is Daughter Cell Size Asymmetry (DCSA), which results in daughter cells of unequal size.

**Fig 1:**
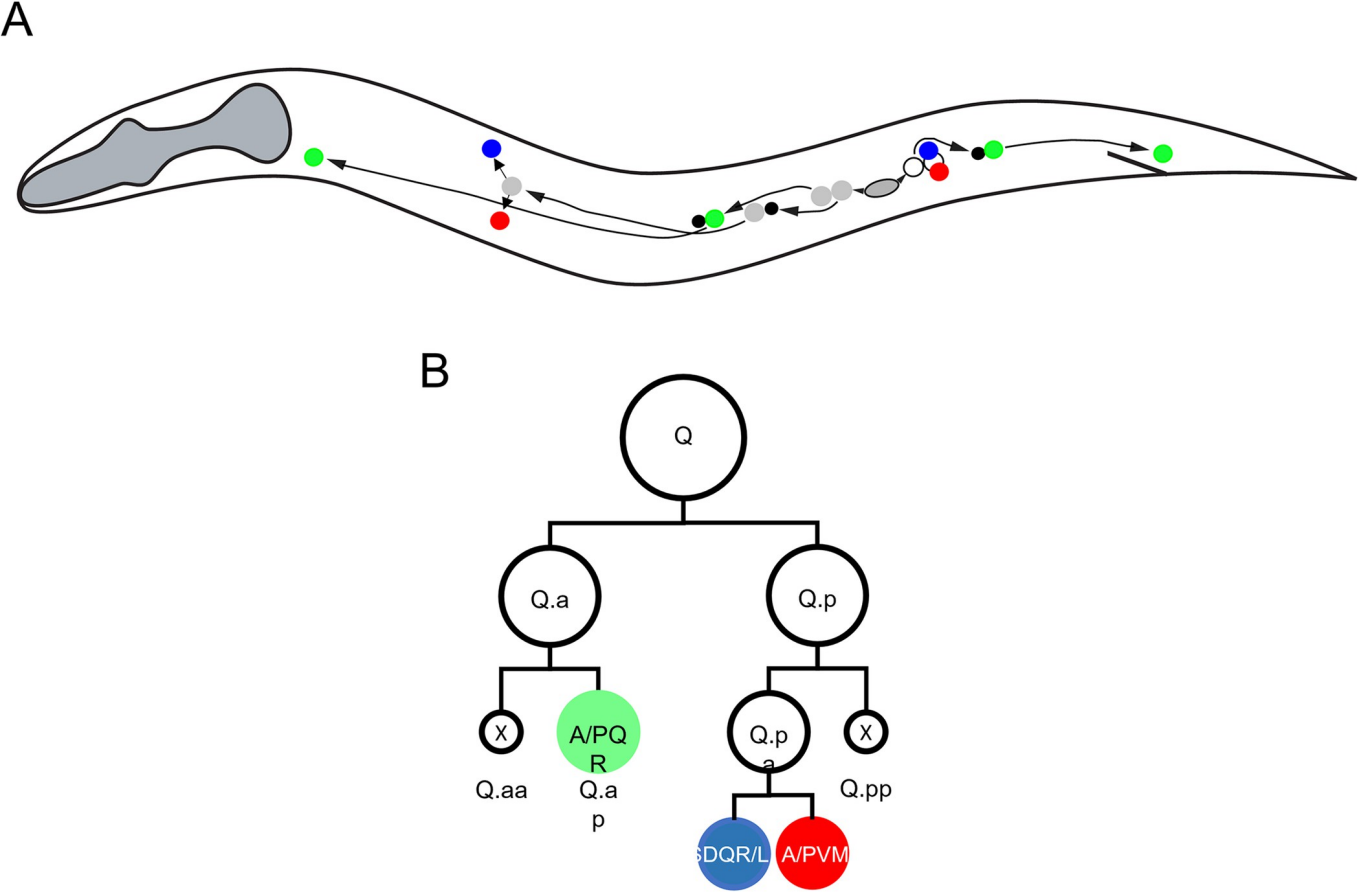
The *C*. *elegans* Q neuroblast divisions. A) The Q lineage migrations. The QL neuroblast and PQR migrate to the posterior while the QR neuroblast and its descendants migrate to the anterior. Solid black daughter cells indicate apoptotic daughter cells after DCSA, and colored cells indicate the final location of the daughter neurons. B) The Q neuroblast lineage. The Q neuroblasts divide to produce Q.a and Q.p, which divide to produce anterior (Q.aa or Q.pa) and posterior (Q.ap or Q.pp) daughter cells. In the Q.a division, the anterior daughter, Q.aa, is smaller and apoptotic. In the Q.p division, the posterior daughter, Q.pp, is smaller and apoptotic. The coloration of the A/PQR, A/PVM and SDQR/L neurons represent the GFP, mCherry, and BFP fluorescence, respectively, of animals bearing the *gmIs81* transgene used to score the numbers and positions of these neurons.

The *C*. *elegans* Q.a and Q.p neuroblast divisions provide examples of different mechanisms of DCSA (Fig 1). The left (QL) and right (QR) neuroblasts each divide to produce anterior (Q.a) and posterior (Q.p) daughter cells. These sister cells both divide to produce a smaller daughter cell that dies and exhibits opposite polarity: the Q.a anterior daughter, Q.aa, is smaller and apoptotic; the Q.p posterior daughter, Q.pp, is smaller and apoptotic. The larger Q.ap becomes the oxygen-sensing neuron A/PQR. The larger Q.pa cell divides to produce the mechanosensory neuron A/PVM and the interneuron SDQR/L. Specifically, the QR lineage generates the AQR, AVM, and SQDR neurons, while the QL lineage generates the PQR, PVM, and SDQL neurons. During this process, the Q lineage cells undergo stereotyped migrations (Fig 1B): QR descendants QR.a, QR.p, and AQR migrate anteriorly, and the QL descendants QL.a and PVQ migrate posteriorly. The A/PVM and SDQR/L neurons undergo short dorsoventral migrations to assume their final positions.

Mutations that disrupt the size asymmetry also disrupt the apoptotic fate of the smaller daughter cell, resulting in extra Q lineage neurons. Different DCSA mechanisms have been reported for the two cells: Q.a divides by a spindle-independent, HAM-1 and myosin-dependent mechanism, whereas Q.p divides by a spindle-dependent, HAM-1 and myosin-independent mechanism [2–4]. Despite these differences, specific proteins are required for both divisions including the PAR-4/PIG-1 kinase pathway and the DEP-containing protein TOE-2 [5, 6].

PIG-1 (*p**ar-**1*-like gene) is the ortholog of the mammalian Maternal Embryonic Leucine-zipper Kinase (MELK) gene and a member of the AMPK family of kinases. MELK has been shown to play a role in a wide range of cellular processes in vertebrates, including cell division, differentiation, death, and survival [7]. In *C*. *elegans*, PIG-1 is involved in numerous asymmetric cell divisions, including the first embryonic division, the NSM neuroblast division, and both the Q.a and Q.p divisions [4, 8, 9]. PIG-1 has been shown to function downstream of a PAR-4/LKB1, STRD-1/STRADα, MOP-25.1,2/ MO25α complex, presumably through phosphorylation of PIG-1 by PAR-4 kinase [5, 8, 10, 11].

TOE-2 was originally defined by its Disheveled Egl-10 Pleckstrin (DEP) domain and multiple predicted Mitogen-Activated Protein Kinase docking sites by bioinformatics and shown biochemically to be a Target of Erk, and subsequently found to regulate both Q.a and Q.p ACD [6, 12]. The DEP domain is a protein-protein interaction domain involved in cell signaling [13]. Using the predicted structure of TOE-2 from AlphaFold in an NCBI VAST search, we found that TOE-2 also has a region with structural similarity to RhoGAP domains [14–16]. The structure of TOE-2 is similar to that of LET-99, a protein that also contains both a DEP domain and a degenerate RhoGAP-like domain and is involved in the first cell and EMS divisions [6, 17, 18]. Based on the homology of the DEP domain and its predicted GAP domain, the closest mammalian homolog of TOE-2 is DEPDC7.

Non-muscle myosin is a central component of the actomyosin network and is involved in a wide variety of processes. The non-muscle myosin NMY-2 plays a key role in establishing the polarity of the first cell division and of the *C*. *elegans* NSM, Q.a and Q.p neuroblast divisions [3, 9, 10, 19]. An interesting complication in generating a unifying model for NMY-2’s role in DCSA is that in these divisions, it localizes to different parts of the progenitor cell relative to the cell’s polarity. In the Q.a division, NMY-2 localizes to the anterior side, which will become the smaller daughter cell fated to die [3]. This is similar to the non-muscle myosin pattern in Drosophila neuroblasts [20, 21]. In the *C*. *elegans* NSM neuroblast, however, NMY-2 localizes to the side that will become the larger surviving NSM daughter cell [10]. This is similar to the *C*. *elegans* first cell division where NMY-2 initially localizes to the side that will produce the larger AB blast cell [22]. However, in Q.p, NMY-2 does not appear to be asymmetric [3]. Despite the differences in NMY-2 asymmetry, PIG-1 regulates DCSA in all of these *C*. *elegans* divisions, and has been shown to regulate NMY-2 localization in the Q.a, NSM and first cell divisions [3, 5, 8, 10]. Considering that, in most of these divisions, NMY-2 has been shown to play a role in furrow positioning independent of spindle positioning, the fact that the localization of NMY-2 is different in these asymmetric divisions is puzzling.

We used endogenously tagged reporters of NMY-2, TOE-2, and PIG-1 to determine their subcellular localization during the Q.a and Q.p cell divisions in order to better characterize their function. We found that TOE-2 was biased toward the side of the cell that would produce the smaller apoptotic cell, whereas PIG-1 was biased toward the side of the cell that would produce the larger surviving cell. We found that, similar to previous reports, NMY-2 localized asymmetrically in Q.a but not Q.p. We used temperature-sensitive *nmy-2* mutants to determine the role of *nmy-2* in these divisions and found that these alleles had only mild effects on the DCSA of the Q.p division and none on the Q.a division. When combined with mutations in *toe-2* or *pig-1*, the *nmy-2* alleles did not significantly alter the Q.a or Q.p DCSA defects of the *toe-2* or *pig-1* mutants but did alter the fate of the Q.a and Q.p daughters in the *toe-2* background and the Q.a daughters in the *pig-1* background. These findings suggest that NMY-2 plays a DCSA-independent role in specifying the fate of the Q.a and Q.p daughter cells.

## Materials and methods

### Strains and genetics

General handling and culture of nematodes were performed as previously described [23]. N2 Bristol was the wild-type strain, and experiments were performed at 20°C unless otherwise noted. Details of the strains used in this study can be found in S1 Table.

The following mutations, integrated arrays and endogenously tagged genes were used:

*LG I*: *nmy-2(ne1490ts*, *ne3409ts)* [24], *nmy-2(cp13)* (*nmy-2*::*gfp+*LoxP), *nmy-2(cp69)* (*nmy-2*::*mKate2+*LoxP) [25]
*LG II*: *toe-2(gm408ok2807)* [6], *toe-2(syb1240)* (*mNeonGreen*::*toe-2*) (this study), *casIs165[egl-17p*::*myr-mCherry; egl-17p*::*mCherry-TEV-S*::*his‐24*, *unc‐76(+)]* [26]
*LG III*: *rdvIs1 [egl-17p*::*mCherry*:*his-24 + egl-17p*::*myristolated mCherry + egl-17p*::*mig-10*::*YFP*::*unc-54 + pRF4]* [3],.
*LG IV*: *pig-1(gm280*, *gm301*, *gm344)* [9], *pig-1(syb2355)* (*pig-1*::*mNeonGreen*) (this study)
*LG X*: *gmIs81 [mec-4p*::*mCherry*, *flp-12p*::*EBFP2*, *gcy-32p*::*gfp*, *egl-17p*::*gfp]* [6]

The C-terminal tagged *pig-1(syb2355)* (*pig-1*::*mNeonGreen*) and N-terminal tagged *toe-2(syb1240)* (*mNeonGreen*::*toe-2*) endogenous insertions were generated by SunyBiotech using CRISPR-Cas9 gene editing.

### Cell count protocol

Worms with the *gmIs81* integrated array were grown on Nematode Growth Media (NGM) seeded with OP50 at 15°C until the plates were populated with gravid adult hermaphrodites. Embryos were then collected after incubating the adults in .75 mL of a solution containing 500mM NaOH and 15% bleach until the adults were mostly dissolved, centrifuged to pellet the embryos which were then washed three times in 1.5 mL of M9, and then plated on standard NGM plates seeded with OP50 and then transferred to 25°C. After 2–3 days, adult and fourth larval stage (L4) worms were transferred to 3–5 μL of 20 mM Sodium Azide in M9 buffer on a 2% agarose pad. Hermaphrodites were scored for the number of observed Q lineage descendants using a Zeiss Axioskop 2 microscope. The number of observed PQR, SDQL and PVM neurons were scored in hermaphrodites with their left, dorsal or ventral side up. While past work has shown that the QR and QL lineages largely behave the same way during DCSA [3], the *gmIs81* marker does not permit reliable scoring of AQR neurons, so the QR descendants were not included in the cell count analysis as they could not be filtered using the method described below.

### Cell count analysis

The frequency of extra or missing cells was calculated for each cell type by dividing the worms with an extra or missing cell by the number of worms scored. The analysis of Q lineage division defects was predicated on the principle that certain cell-fate changes produce unique patterns that cannot be produced by a Q.aa or Q.pp cell that is normally fated to die surviving and adopting the fate of its sister cell or niece (S1 Fig). For instance, occasionally Q.a will adopt the fate of Q.p leading to a missing A/PQR and extra SDQL/R or A/PVM neurons. Another possibility is a progenitor may fail to divide, leading to the loss of its descendants. To eliminate lineages with these types of defects, each pattern of potential cell counts was analyzed to determine whether the production of extra neurons would likely result from a failure in apoptosis and transformation into its sister cell or niece. Only those lineages were counted in the filtered Q.a and Q.p results. The criteria for excluding a pattern were if a cell was missing or if there were three or more of any cell type. These patterns were arranged into defect categories corresponding with which daughter cell survived. In the final analysis, we used two defect categories, QL.a and QL.p, with the patterns and categories described in Table 1. The frequency of each category was determined by the number of worms exhibiting that category of defect divided by the number of worms scored for that lineage. Specifically, the frequency of QL.p defects was calculated as (# QL.p defective)/(# QL.p defective + # QL.p normal), while the frequency of QL.a defects was calculated as (# QL.a defective)/(# QL.a defective+ # QL.a normal).

**Table 1:** QL lineage cell count defect filtering.

# CELLS ON LEFT SIDE			DEFECT PRESENT?	
PQR	SDQL	PVM	QL.a	QL.p
**1**	1	1	No	No
**1**	1	2	No	Yes
**1**	2	1	No	Yes
**1**	2	2	No	Yes
**2**	1	1	Yes	No
**2**	1	2	Yes	Yes
**2**	2	1	Yes	Yes
**2**	2	2	Yes	Yes

The filtering method that was used to determine what cell count patterns corresponded to each defect type for the left side Q lineage neurons while excluding all cases requiring a cell fate transformation beyond the survival of the QL.aa or QL.pp cell.

### Imaging

Worms with the *gmIs81* integrated array were grown on NGM seeded with OP50 at 15°C until the plates had a large number of embryos and larvae. The plates were then put at 25°C for 4 hours. Worms were then washed off the plates with M9 and transferred to microcentrifuge tubes. They were then spun in a tabletop centrifuge for less than 6 seconds to pellet the larger, adult worms. The supernatant was then transferred using a glass pipette to a fresh microcentrifuge tube. This was then spun for 30 seconds and the supernatant was removed until only ~100 μL remained. 2 μL of 1M sodium azide was added, and the tube was briefly vortexed and spun for 30 seconds. All but ~10 μL of supernatant was removed, and using a glass pipette, the pellet and remaining M9 + sodium azide was transferred to a 2% agarose slide. To determine the sizes of the daughter cells and the number of Q-derived neurons, the worms were imaged using an Axio Observer Z1 microscope.

For time-lapse imaging, worms were prepared as above except there was no 25°C step, and 1.25 mM levamisole was used to anesthetize the worms instead of 20 mM sodium azide. Time-lapse images of Q neuroblast divisions were captured with seven plane Z-stacks (Z-step: 0.5 μm) in 30-second intervals on a spinning-disk (CSU-X1; Yokogawa) confocal microscope. Images were captured using an EM CCD camera (Evolve; Photometrics) and SlideBook software (Intelligent Imaging Innovations).

### Image analysis

Image analysis was performed using the FIJI package for ImageJ [27]. To measure DCSA, the outlines of recently divided Q.p or Q.a daughters in worms containing the *gmIs81* marker were traced using the lasso select tool in ImageJ, and the area was measured for each cell to determine the ratio. Each cell was measured three times and the final ratio was calculated using the average of the three measurements.

To measure the localization of the endogenously tagged reporters, we identified time points for metaphase, anaphase, telophase and cytokinesis in the time-lapse images and created sum Z-projections of the slices containing the best cross-section of the dividing cell. The slice number varied as slight movements of the worm can cause the Q.a and Q.p to shift or tilt during imaging such that capturing the full perimeter of all imaged cells with a single stack size is not feasible. We then performed line scans around the cortex, using the segmented line and plot profile tools in FIJI, measuring the intensity of the endogenously tagged protein in the 488 nm channel and myristoylated mCherry in the 561 nm channel across 3 pixels every 1/6 microns. A line was drawn through either the metaphase plate or cleavage furrow and measurements started at a point where that line intersected the membrane and followed the entire cell cortex as marked by the myristoylated mCherry. The other point intersecting the line of the metaphase plate or cleavage furrow was then marked as were the anterior and posterior sides of the furrow. We also established the background fluorescence levels by measuring the average intensity in each channel of a section of the body cavity that expressed neither reporter using the same z-projection as the measurement.

For analysis, the background values were subtracted from the intensity values of all measured points. To compensate for both variations in z-stack size and potential variations in membrane density, we normalized the measurements within each time point for each cell by dividing the intensity at 488 nm by the intensity at 561 nm for each point, which we refer to as the normalized intensity ratio. These values were used for a basic comparison by determining the ratio between the average normalized intensity of the anterior and posterior of each cell at each time point. The average of these ratios and standard deviations for each phase and each strain are found in S2 Table.

### Line-scan modelling

To model the line-scan data, the normalized intensity ratio for each point was paired with information about the relevant variables. To compare cells of different sizes, we determined the Normalized Distance by dividing the distance from the start of the measurement by the total distance measured. We also determined the normalized distance between each point and the nearest metaphase plate or cleavage furrow point to determine the Furrow Distance. Each point was then paired with its normalized intensity ratio, the cell type, the phase of the division, and whether it was anterior or posterior to the division plane.

The line-scan information was used to construct Generalized Mixed Linear Models (GLMMs) using the MCMCglmm package in R [28, 29]. We chose to use GLMMs because they permit flexible analysis of nonnormal data with random effects [30]. We used Log base 2 of the normalized intensity ratio as the dependent variable, the specific measurement as a random variable, and the Cell Type, the Phase, Anterior vs Posterior, and the Furrow Distance as fixed variables. We also added the interaction terms of Phase and Anterior vs Posterior as well as Phase and Furrow Distance to account for changes in their effects in different phases. The resulting models allowed us to estimate the effect size of each variable.

In order to determine whether there was a significant difference between the Anterior and Posterior effects during each phase, we used the emmeans R package to calculate the estimated marginal means for the Phase and Anterior vs Posterior interaction term [31]. Estimated marginal means are the marginal means of model predictions over the grid containing all factor combinations. The estimated marginal mean for a specific variable can be viewed as an estimate of the effect size and direction of a specific variable on the model after accounting for all other variables [32]. Using the emmeans pairs function to perform pairwise comparisons between the different estimated effect sizes, we found the difference between the estimated effect sizes for each pair of interaction pairs of Phase and Posterior vs Anterior. The estimated difference between the Anterior and Posterior in each phase is then used to calculate a z-ratio and Tukey adjusted p value. The sign of the estimated difference indicates the direction, with positive values indicating a stronger Posterior effect and negative values indicating a stronger Anterior effect. Plots were made using the gather_emmeans_draws function in the tidybayes R package to generate draws from the marginal posterior distributions of the models and the ggplot2 R package to generate the plots from those draws [33, 34].

### Statistical analysis

Statistical analysis was performed using the two-sample Z-test for proportions using the Benjamani-Hochberg procedure to correct for false discovery for extra cell count scoring and the Kruskal-Wallace H test for cell size ratios.

## Results

### NMY-2, TOE-2, and PIG-1 are asymmetrically distributed during the divisions of Q.a and Q.p neuroblasts

The *C*. *elegans* Q lineage has been a model for studying ACD. The left and right Q cells each undergo a series of divisions along the anterior-posterior axis (A-P) to generate three neurons and two cells fated to die [35]. The Q daughters, Q.a and Q.p, each divide to generate daughter cells that are asymmetric in size and fate (Fig 1). Q.a divides to generate a smaller anterior daughter cell that dies and a larger posterior daughter that survives and differentiates into an A/PQR oxygen-sensing neuron. Q.p divides with the opposite polarity to generate a smaller posterior daughter that dies and a larger anterior daughter that survives and divides to generate the A/PVM mechanosensory neuron and the SDQR/L interneuron. Because the right and left Q cells and their descendants migrate in opposite directions, the neurons on the right side (AQR, AVM and SDQR) are in positions anterior to those on the left side (PQR, PVM and SDQL).

**Fig 2:**
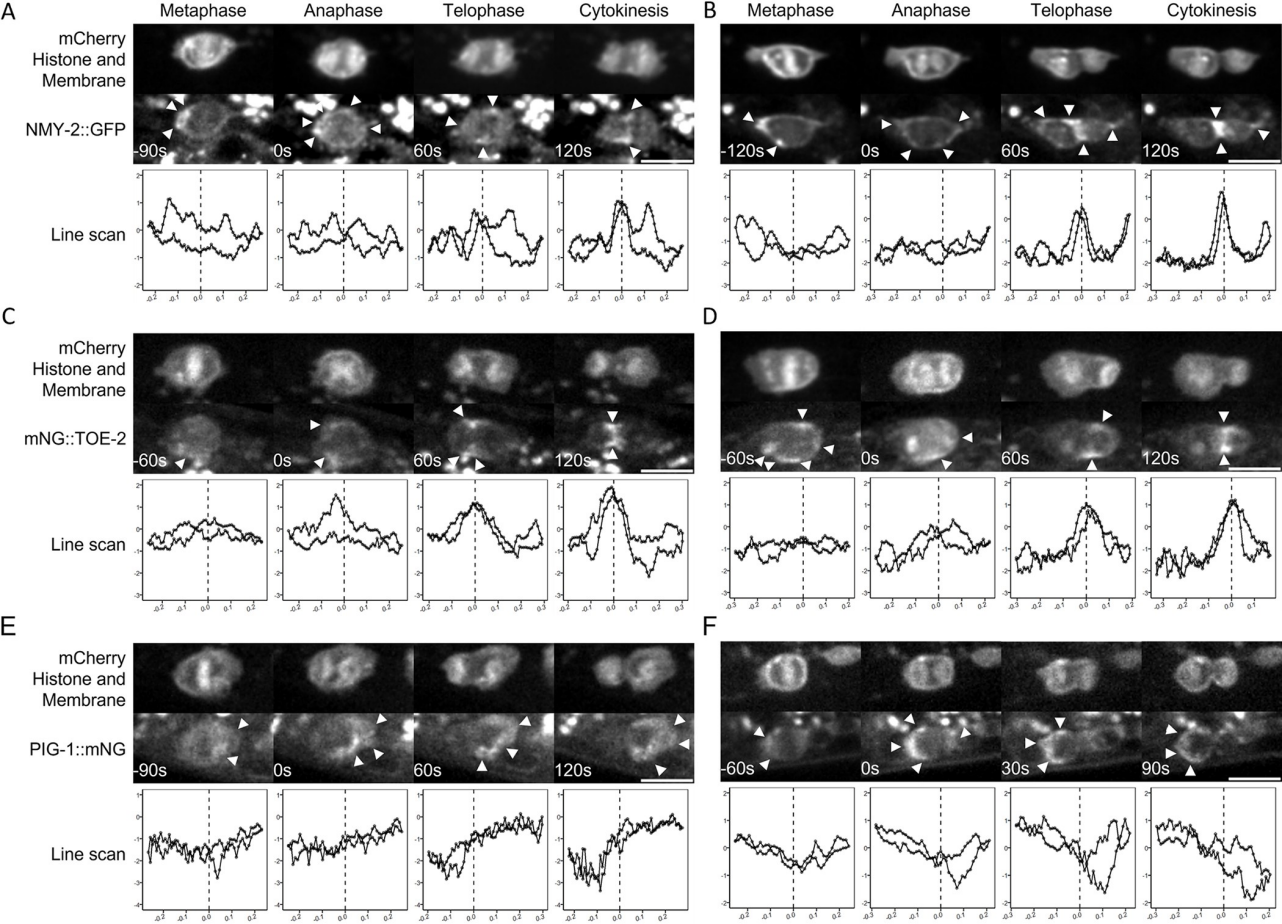
Time-lapse images of endogenously tagged DCSA proteins in the Q.a and Q.p divisions. A-F) Time-lapse imaging with line scan profiles of QR.a and QL.p divisions in A,B) NMY-2::GFP, C, D) mNeonGreen::TOE-2, and E, F) PIG-1::mNeonGreen. Arrowheads indicate areas of higher GFP or mNeonGreen signal, and scale bars are 5 μm. Line scan profiles show the normalized intensity ratios (Log 2) on the y-axis and normalized distance along the AP axis on the x-axis. The distance is defined in relation to the division plane or cleavage furrow, indicated by the dotted line at zero, with negative values indicating the anterior and positive values indicating the posterior.

The non-muscle myosin NMY-2, the AMPK-family kinase PIG-1, and the DEPDC7 homolog TOE-2 all regulate the asymmetry of the Q.a and Q.p divisions [3, 6, 9]. To better understand the roles of these molecules in asymmetric cell division, we took time-lapse images of the Q.a and Q.p divisions in strains containing endogenously tagged NMY-2::GFP, PIG-1::mNeonGreen, or mNeonGreen::TOE-2 (Fig 2). We then measured the intensity of the GFP or mNeonGreen at the cortex and normalized it to an mCherry cortical marker. Taking the average normalized intensity of the posterior and anterior sides of the cells (S2 Table), we observed possible asymmetry of NMY-2::GFP (Fig 2A and 2B) and mNeonGreen::TOE-2 (Fig 2C and 2D) towards the side of Q.a or Q.p that will produce the daughter cell fated to die, and strong asymmetry of PIG-1::mNeonGreen towards the side that will produce the daughter cell that survives (Fig 2E and 2F). It is noteworthy that the PIG-1::mNeonGreen strain had a cell fate defect (6.6% extra Q.p neurons N = 97) indicating that the tag affects *pig-1* function. This is a weak phenotype compared to the *pig-1* null mutants that, under the same conditions, exhibited a greater than tenfold higher frequency of Q.p defects as well as exhibiting a low frequency of Q.a defects (discussed below).

Because both NMY-2 and TOE-2 localize to the cleavage furrow during anaphase and telophase, the ratio of the average intensity between the posterior and anterior could be misleading as identical levels of the protein flanking the furrow combined with the size asymmetry of the daughter cells would result in the smaller side having a higher average intensity. To mitigate this problem, we performed line scans around the cortex and annotated each measured point on the line scan with the intensity normalized by taking the ratio of the intensity of the GFP signal and the intensity of the mCherry cortical marker, the distance from the division plane normalized to the circumference of the cell, the phase of the division, and whether the point was anterior or posterior to the division plane.

We used this information to construct two Generalized Linear Mixed Models (GLMMs) for each reporter, one for Q.a and one for Q.p. GLMMs permit the modeling of data with a mixture of random and fixed variables and nonnormal distributions and variances [29]. All of the models used the log base 2 of the normalized intensity ratio as the dependent variable, the specific cell measurement as a random variable, and the phase, furrow distance and whether it was anterior or posterior, as fixed variables. We also included interaction terms between the phase and anterior or posterior as well as phase and furrow distance. Interaction terms account for the difference in the effect of one variable based on the value of another variable. In this model, these interaction terms account for the fact that the distribution of the protein with respect to both the A-P bias and proximity to the furrow can vary between different phases. The resulting models allowed us to estimate the effect size of each variable, and, most importantly, the interaction terms allowed us to compare the relative levels of the reporter at the anterior and posterior within each phase after accounting for the other variables. The resulting models’ estimates and parameters can be found in S3 Table.

**Fig 3:**
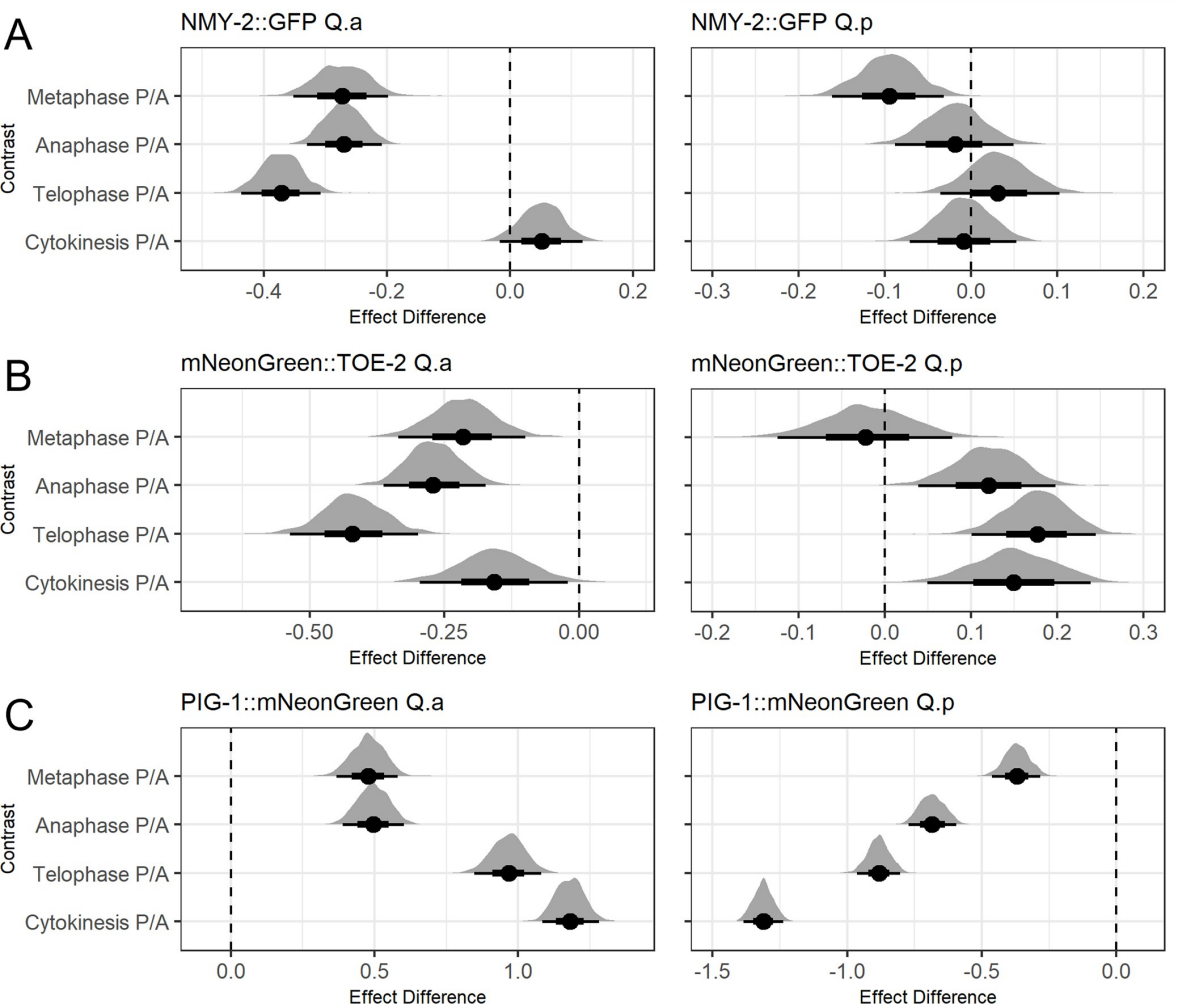
Line scan modelling analysis of endogenous reporters shows asymmetry in the Q.a and Q.p divisions. A-C) Modelling analysis comparing anterior and posterior effect in Q.a and Q.p in Metaphase, Anaphase, Telophase and Cytokinesis for A) NMY-2::GFP, B) mNeonGreen::TOE-2, and C) PIG-1::mNeonGreen. The black bars represent the confidence intervals, while the distributions represent the frequency of draws of that value. Negative values indicate a greater anterior effect, positive values indicate a greater posterior effect. Full statistical analysis and model details can be found in S1 and S2 Tables.

Using our models, we found that there was significantly more NMY-2::GFP at the anterior of Q.a during metaphase, anaphase, and telophase. During cytokinesis, NMY-2 was not asymmetric and localized to the cleavage furrow (Fig 3A and S2 Table). Our model showed no significant Q.p asymmetry in NMY-2::GFP localization over the course of its division (Fig 3A and S2 Table). These results are in agreement with those previously reported using an NMY-2::GFP transgene [3].

Our model for the distribution of mNeonGreen::TOE-2 in Q.a was similar to that of NMY-2::GFP: it had a greater anterior distribution throughout the Q.a division though, unlike NMY-2::GFP, mNeonGreen::TOE-2 remained asymmetric during cytokinesis (Fig 3B and S2 Table). TOE-2 localized to the posterior of Q.p in telophase and cytokinesis (Fig 3B and S2 Table). We were, however, unable to determine whether the two tagged proteins colocalize in the Q lineage because the GFP and mCherry markers we used to detect these cells interfered with our ability to detect NMY-2::GFP or mNeonGreen::TOE-2, respectively.

The distribution of TOE-2 reported here differed from previous descriptions of GFP-tagged transgenes. A GFP-tagged *toe-2* cDNA expressed from an *egl-17* promoter accumulated in the nuclei of interphase cells [6], but we detected no nuclear TOE-2 with the endogenously tagged gene.

We also observed that mNeonGreen::TOE-2 localized to the junction between the germline progenitors Z2 and Z3 and continued to localize to the apical surface of the germline cells through all stages of development (S2 Fig). While the constraints of Q-cell imaging with the markers available to us did not permit reliable detection of colocalization between TOE-2 and NMY-2 in the Q lineage, we did find that mNeonGreen::TOE-2 and NMY-2::mKate2 colocalized in the germline (S3 Fig). NMY-2 localizes to the lateral membranes that separate the germline nuclei and accumulates at the ring channels that form the pores that connect the germ cells to a central canal, the rachis [36]. The presence of TOE-2 suggests a possible role for it in the ring channels as well.

Our models showed that PIG-1::mNeonGreen was much more asymmetric than either NMY-2::GFP or mNeonGreen::TOE-2, localizing to the posterior of Q.a and the anterior of Q.p during mitosis. For both Q.a and Q.p, the PIG-1::mNeonGreen asymmetry increased as the cells progressed through mitosis (Fig 3C).

### Temperature-sensitive *nmy-2* mutants reveal a role in Q lineage ACD

**Fig 4:**
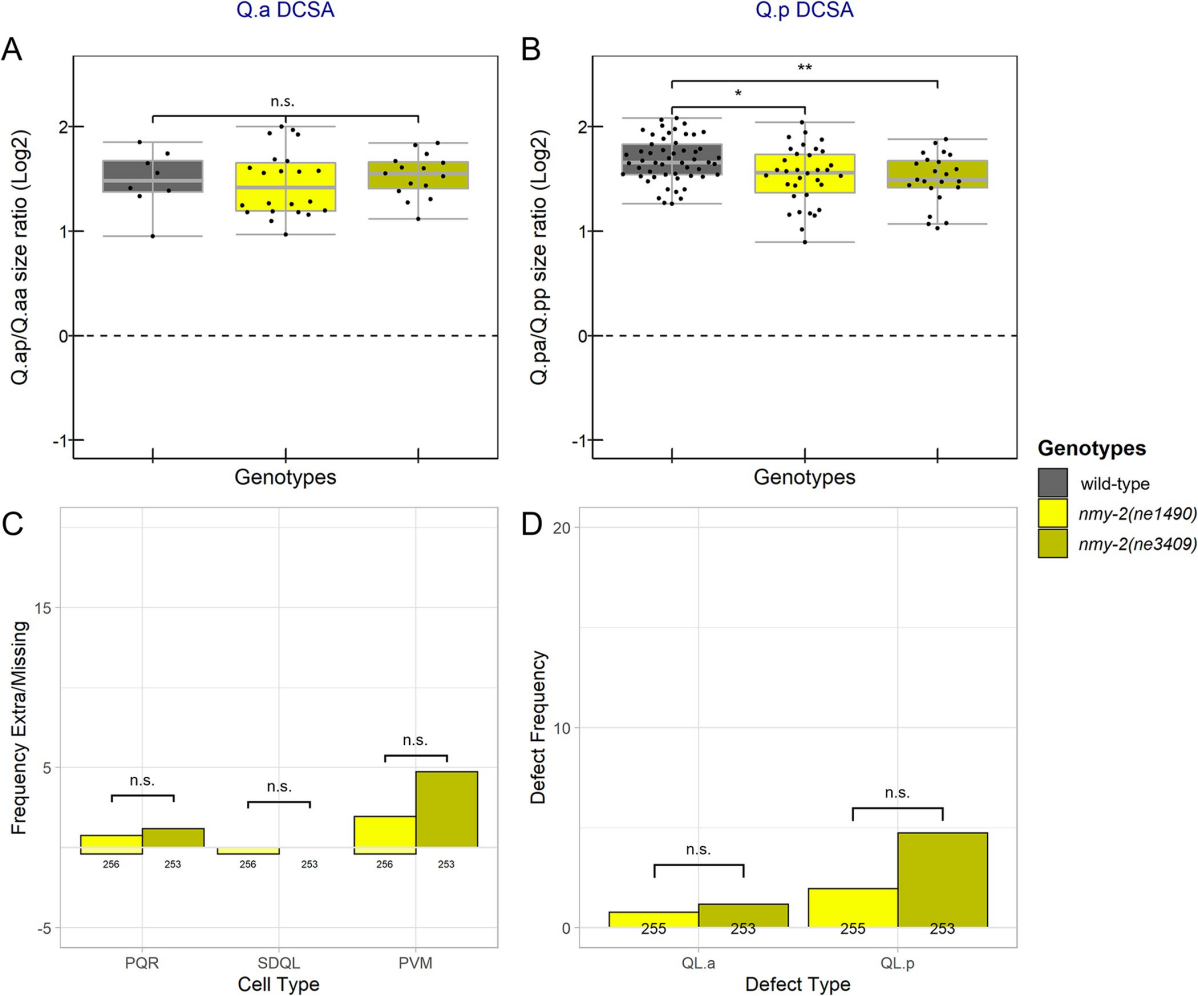
DCSA and cell-fate defects in temperature-sensitive *nmy-2* mutants. A,B) Box plots of the area ratios of A) Q.ap/Q.aa and B) Q.pa/Q.pp divisions in control and *nmy-2*(ts) mutants. C) Frequency of extra (positive y-axis) and missing (negative y-axis) Q lineage cells. D) Frequency of extra cell defects that could be explained by survival of QL.aa or QL.pp. (C, D) Below each bar are the number of lineages scored. *: P<0.05, **: P<0.01, ***: P<0.001, n.s.: P>0.05.

To further characterize the role of NMY-2 in the Q lineage divisions, we asked whether the two temperature-sensitive *nmy-2* mutants, *nmy-2(ne1490ts)* and *nmy-2(ne3409ts)*, had altered Q.a and Q.p DCSA when shifted to 25°C four hours before imaging. While both alleles are lethal to early embryos at the restrictive temperature [24, 37], we found that when shifted to the restrictive temperature as either late-stage embryos or L1 larvae, the mutants grew into adults. We detected a significant decrease in Q.p DCSA in both *nmy-2(ne3409ts)* (P<0.01) and *nmy-2(ne1490ts)* mutants (P<0.05) compared to the control. Neither mutant allele had a significant effect on Q.a DCSA (Fig 4A and 4B). While performing these experiments we observed occasional persistent intracellular bridges between the daughter cells of both Q.a and Q.p in strains containing the *nmy-2(ts)* alleles suggesting potential defects in abscission (S4 Fig).

We also assessed the fates of the QL cell descendants in the mutants by counting the number of PQR, SDQL, and PVM neurons in adult hermaphrodites that had been shifted to 25°C during embryonic development. To determine the number of these cells, we used the *gmIs81* reporter, which labels each cell type with a different fluorescent marker [6]. We did not count QR descendants because we could not reliably distinguish AQRs from other neurons expressing GFP in the head. The two *nmy-2* mutants had a low frequency of extra PQR and PVM cells and *nmy-2(ne1490*ts*)* had a low frequency of missing PQR, SDQL, and PVM cells (Fig 4C). The wild-type control, not shown, had no extra or missing cells (N = 133).

Some of these phenotypes are difficult to interpret and may result from a failure of progenitor cells to divide or cell-fate transformations earlier in the lineage. We propose several lineage defects that can explain these phenotypes in S1 Fig. For example, worms that lack Q.p descendants but have two or more Q.a descendants potentially represent Q.p to Q.a transformations [6]. Because of these complications, we filtered the cell counts to remove all instances with either a missing cell or three or more of a given cell type. These filtered cell counts were then grouped based on whether they had QL.a or QL.p defects (Table 1). Both temperature-sensitive *nmy-2* mutants had increased frequencies of QL.a and QL.p defects compared to wild-type (Fig 4D). There was no significant difference in the frequency of QL.a or QL.p defects between *nmy-2(ne1490*ts*)* and *nmy-2(ne3409*ts*)* raised at the nonpermissive temperature (Fig 4C and 4D).

### Temperature-sensitive *nmy-2* alleles alter *toe-2* mutant Q lineage cell-fate defects but not Q.a or Q.p DCSA

**Fig 5:**
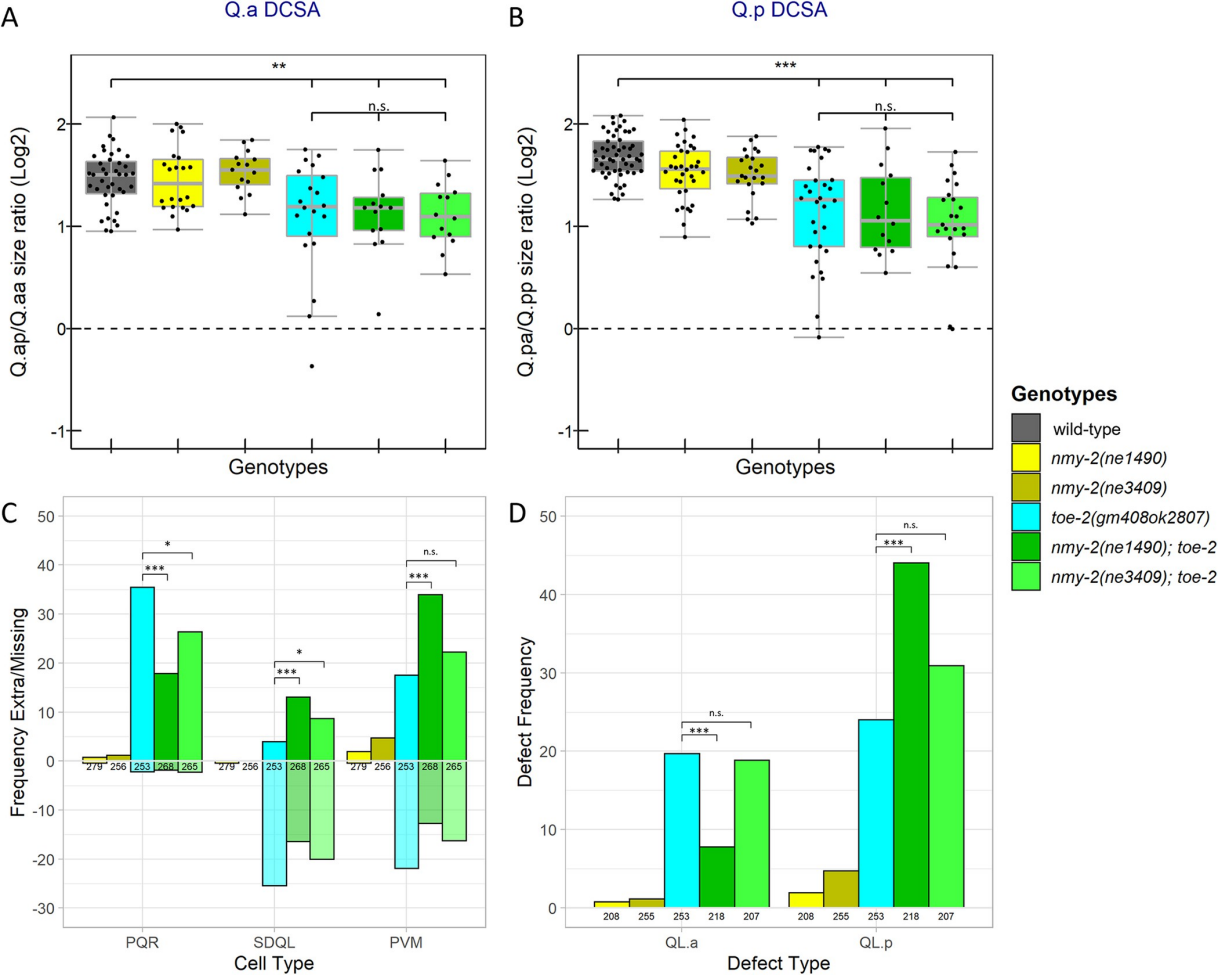
Temperature-sensitive *nmy-2* mutants enhance *toe-2* Q lineage cell fate but not DCSA defects. A, B) Box plots of area ratios of A) Q.ap/Q.aa and B) Q.pa/Q.pp. C) Frequency of extra (positive y-axis) and missing (negative y-axis) QL lineage cells. D) Frequency of extra cell defects that could be explained by the survival of QL.aa or QL.pp with no other cell-fate transformations. (C, D) Below each bar are the number of lineages scored. *: P<0.05, **: P<0.01, ***: P<0.001, n.s.: P>0.05.

The similar distributions of NMY-2 and TOE-2 during the Q.a divisions suggest that they may function together in the Q lineage. We constructed double mutants with the presumptive null *toe-2(gm408ok2807)* allele and each temperature-sensitive *nmy-2* allele to determine if the two genes function together or in parallel. Consistent with the two genes acting together, no significant DCSA differences existed between the *toe-2* single mutant and either *toe-2; nmy-2* double mutant (Fig 5A and 5B). We note that contrary to previous findings from our lab [6] *toe-2(gm408ok2807)* exhibited a Q.p DCSA defect (Fig 5B). This difference may result from differences in the reporters used in the two studies. Because the *nmy-2* mutants do not alter Q.a DCSA, the lack of a *toe-2* enhancement is difficult to interpret, but because the mutants do alter Q.p DCSA, the lack of enhancement suggests that *nmy-2* and *toe-2* function together to regulate the size asymmetry of this division.

Our observations of cell fate in the single and double mutants were more complicated. When compared to *toe-2(gm408ok2807)*, the *nmy-2(ne1490*ts*); toe-2* strain had a significant (p<0.001) increase in the frequency of extra SDQL and PVM neurons and a decrease in the frequency of extra PQR neurons (Fig 5C). After filtering, there was a similar increase in QL.p defects and decrease in QL.a defects in the double mutants (Fig 5D). The *nmy-2(ne3409ts); toe-2* strain had weaker effects: a significant (P<0.05) increase in the frequency of extra SDQL cells and a significant decrease in the frequency of extra PQR cells (P<0.05) (Fig 5C). However, the *nmy-2(ne3409ts); toe-2* strain was not significantly different from *toe-2(gm408ok2807)* in the frequency of QL.a or QL.p defects after filtering (Fig 5D).

### Temperature-sensitive *nmy-2* alleles suppress *pig-1(gm301)* Q.a cell fate defects while not significantly altering Q.a DCSA

**Fig 6:**
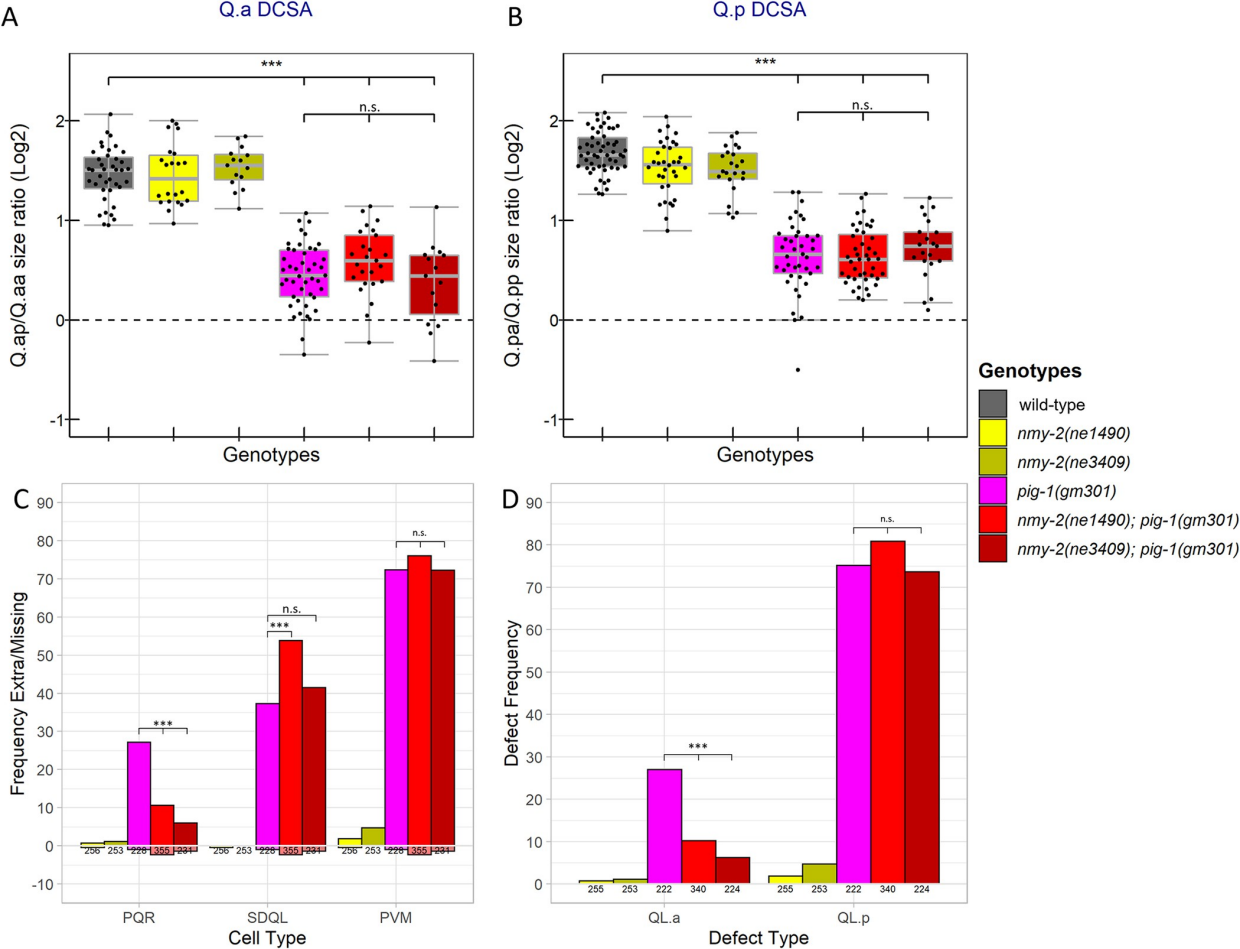
Temperature-sensitive *nmy-2* alleles suppress *pig-1(gm301)* Q.a cell fate defects while not significantly altering Q.a DCSA. A,B) Box plots of the area ratios of A) Q.ap/Q.aa and B) Q.pa/Q.pp divisions. C) Frequency of extra (positive y-axis) and missing (negative y-axis) QL lineage cells. D) Frequency of extra cell defects that could be explained by survival of QL.aa or QL.pp. (C, D) Below each bar are the number of lineages scored. *: P<0.05, **: P<0.01, ***: P<0.001, n.s.: P>0.05.

Our finding that PIG-1 and NMY-2 localized to opposite sides of Q.a suggests that these two molecules play different roles in these cells. To determine how *pig-1* and *nmy-2* interact, we constructed double mutants with *pig-1(gm301)* and each of the temperature-sensitive *nmy-2* alleles and scored the single and double mutants for Q.a and Q.p DCSA defects and the presence or absence of Q-lineage neurons. There were no significant differences in DCSA of either Q.a or Q.p between *pig-1* single and *nmy-2; pig-1* double mutants (Fig 6A and 6B). Both double mutant strains had a significant decrease in the frequency of extra PQR neurons and QL.a-specific defects when raised at the nonpermissive temperature of 25°C. (Fig 6C and 6D). The *pig-1* single and the *nmy-2; pig-1* double mutants displayed similar Q.p defects.

The significant increase in SDQLs in the *nmy-2(ne1490ts); pig-1(gm301)* strain compared to the *pig-1(gm301)* strain is potentially due to what is referred to by Mishra et. Al. [38] as an increase in mitotic potential (Fig 6C). Specifically, in instances where QL.pp survives in *pig-1(gm301)*, it divides 46.2% of the time, while in *nmy-2(ne1490); pig-1(gm301)* it divides significantly (P<0.001) more frequently, 63.8% of the time (S4 Table).

## Discussion

### PIG-1 is asymmetric in Q.a and Q.p

Previous studies using a *Ppig-1*::*pig-1*::*gfp* transgene showed that PIG-1::GFP localized to the cortex and centrosomes, but did not report any asymmetry in the Q lineage [5]. The endogenously tagged PIG-1::mNeonGreen showed cortical localization as well as a clear asymmetry towards the side of the neuroblast that would produce the daughter cells fated to live during both the Q.a and Q.p divisions.

Using double mutant strains of *pig-1* and the temperature-sensitive *nmy-2* alleles, we observed no significant change in surviving QL.pp cells compared to *pig-1* on its own. We observed an increased frequency of QL.p lineages that produced both extra SDQL and PVM neurons as opposed to just one extra neuron and interpret this as an increase in mitotic potential. By contrast, there was a significant decrease in the frequency of surviving QL.aa cells in both *nmy-2(ne1490*ts*); pig-1* and *nmy-2(ne3409*ts*); pig-1* when compared to *pig-1* on its own. This suggests that *nmy-2* functions downstream of *pig-1* in the Q lineage.

In support of this model, *nmy-2* functions downstream of *pig-1* in the *C*. *elegans* NSM neuroblast division [10]. The authors showed that NMY-2 lost its cortical asymmetry in the NSM and identified two phosphorylation sites on NMY-2 that were partially dependent on PIG-1 for phosphorylation. They also found that phosphomimetic NMY-2 was able to partially rescue the loss of PIG-1 in the NSM division.

An interesting set of future experiments would be to determine if PIG-1 has the same pattern of cortical asymmetry towards the larger side of other asymmetric divisions throughout *C*. *elegans* development, as we know from the difference between the localization of NMY-2 in the NSM neuroblast division and the Q lineage divisions that NMY-2’s localization pattern relative to the size of the daughter cell is not constant.

### NMY-2 is asymmetric in Q.a and functions in Q.p DCSA

Our results for NMY-2 are interesting in that they do not align fully with previously reported findings. In particular, Ou et al. performed Chromophore Assisted Laser Inactivation (CALI) experiments where they inactivated a GFP::NMY-2 transgene at the anterior of Q.a or the posterior of Q.p during late anaphase [3]. In Q.a, they saw that this perturbation caused an attenuation, loss, or reversal of DCSA and an increased rate of survival and differentiation of Q.aa. However, they observed no change in Q.p DCSA [3]. This led them to conclude that *nmy-2* primarily regulates Q.a DCSA.

By contrast, our experiments with temperature-sensitive *nmy-2* mutants showed no significant change in Q.a DCSA and a significant reduction in Q.p DCSA. Supporting this observation, we observed a higher frequency of extra Q.p lineage cells than Q.a lineage cells in the mutant strains.

What could explain these differences? A possible confounding factor in the CALI experiments is the presence of endogenous NMY-2. Indeed, a comparison of the *zuIs45* integrated array used in the CALI experiments with endogenously tagged *nmy-2* showed that *zuIs45* had lower levels of expression than the endogenously tagged NMY-2, suggesting that there is more endogenous NMY-2 than tagged NMY-2 in the strain used for the CALI experiment [25]. Whereas the CALI experiments would have an effect by reducing the levels of NMY-2 in the inactivated region, the expression of endogenous, untagged NMY-2 makes it likely that NMY-2 activity persisted in the irradiated areas, complicating the interpretation of these experiments. In particular, the difference between the Q.p results could easily be explained by the presence of endogenous NMY-2. The difference between Q.a results is harder to explain but could be due to a difference between a general and local disruption of NMY-2 activity.

In Drosophila neuroblasts, as in *C*. *elegans* Q.a, more non-muscle myosin localizes to the side that will produce the smaller cell. In the Drosophila neuroblast, this localization is thought to produce cortical contractions that produce a cortical flow that is required to correctly distribute polarity proteins [39, 40]. In the *C*. *elegans* NSM neuroblast and the first cell division, NMY-2 localizes to the opposite side of the dividing cell, the side that will produce the larger daughter cell [20]. In the NSM neuroblast division, the *nmy-2(ne3409ts)* mutation results in a complete loss of DCSA. The authors proposed that NMY-2 creates cortical flows that are required to establish the gradient of cell-fate determinants and increase cortical contractility on the ventral side, which will produce the larger daughter cell [10]. This model contrasts with the models of cortical contractility on the side that produces the smaller daughter cell. Further experiments are needed to determine which model would be correct in the context of the Q lineage.

### NMY-2 has a role in Q-lineage fate determination that is independent of its role in DCSA

The temperature-sensitive *nmy-2* alleles caused defects in cell-fate determination in both Q.a and Q.p despite having no or a modest impact, respectively, on the DCSA of those divisions. These findings suggest that NMY-2 has a DCSA-independent role in cell fate determination in the Q.a and Q.p divisions. The finding that NMY-2 regulates cell fate has also been observed in the NSM neuroblast division. Besides eliminating DCSA, the *nmy-2(ne3409ts)* mutation disrupted the gradient of CES-1, a Snail-like transcription factor and cell-fate determinant [10].

We observed that the endogenously tagged mNeonGreen::TOE-2 and NMY-2::GFP reporters exhibited similar localization patterns in the Q.a division, with both being biased towards the side that will produce the daughter cell fated to die as well as to the cleavage furrow. This localization suggests that they may have related functions. Consistent with this possibility, double mutants of *toe-2* and the temperature-sensitive alleles of *nmy-2* exhibit no increase in DCSA in either the Q.a or Q.p divisions when compared to *toe-2* on its own but did have differences in specifying the fate of their descendants. Only the *nmy-2(ne1490ts)* mutation interacted with *toe-2*, increasing the frequency of the *toe-2* mutant QL.p defects while decreasing the frequency of the QL.a defects, while the *nmy-2(ne3409ts); toe-2* double mutant and single *toe-2* mutant defects did not significantly differ. The interaction between *nmy-2(ne1490ts)* and *toe-2* is consistent with a role for NMY-2 in regulating cell fate independent of a role in DCSA in both Q.a and Q.p. A DCSA-independent role is further supported by the finding that both *nmy-2* mutations suppressed the frequency of QL.a defects in *pig-1(gm301)* mutants without significantly altering *pig-1* mutant Q.a DCSA.

A possible mechanism for NMY-2’s function in specifying fate in the Q lineage is to distribute cell fate determinants, as was shown for CES-1 in the NSM neuroblast [10]. This has also been observed in Drosophila neuroblasts where non-muscle myosin is required for the basal distribution of two cell-fate determinants, Prospero and Numb [41]. Another possible explanation would be an indirect effect wherein the *nmy-2* mutants may have defects that slow cytokinesis or abscission, potentially resulting in cell fate determinants losing asymmetry due to a persisting connection between the daughter cells. This is supported by the observation of occasional persistent intercellular bridges between the daughter cells of both Q.a and Q.p in strains containing the *nmy-2(ts)* alleles (S4 Fig) as well as the fact that the original characterization of the *nmy-2(ts)* alleles reported that the cleavage furrow of the first embryonic division would halt or regress when the embryo was shifted to the non-permissive temperature [24].

Further experiments to determine how the loss of these three genes in the Q lineage influences the localization of the others, as well as other cell fate factors, will be an important step in understanding their DCSA-dependent and independent roles in cell fate specification.

## Supporting information

S1 Fig Possible lineages for Q lineage specification defects. A) The wild-type Q lineage. B) A Q.a division defect where the Q.aa fails to die and adopts the Q.ap fate as an A/PQR neuron. C) A Q.p division defect where the Q.pp fails to die and adopts the Q.pa fate. D) A Q.p division defect where the Q.pp fails to die but does not divide and adopts an SDQR/L or A/PVM fate. E) A Q division defect where Q.p adopts the fate of Q.a. This results in an extra A/PQR and an absence of the Q.p descendants. F) A Q division defect, where the Q.a adopts the fate of Q.p. This results in a duplication of the SDQR/L and A/PVM neurons and an absence of the A/PQR. The Q.p transformation in E and the Q.a transformation in F may also display the defects shown in B, and C and D respectively, resulting in three or more neurons that express a specific fate.
(TIF)

S2 Fig Confocal imaging of endogenously tagged TOE-2 localizing to the apical surface of the germ cells. A) mNeonGreen::TOE-2 in a first larval (L1) stage hermaphrodite. Closed arrowhead indicates mNeonGreen::TOE-2 at the point of contact between Z2 and Z3. B) mNeonGreen::TOE-2 in an L3 stage hermaphrodite. Closed arrowheads indicate localization of mNeonGreen::TOE-2 to the apical surface of the germline cells. Open arrowheads indicate mNeonGreen::TOE-2 localization to unknown cells near the vulva. C) mNeonGreen::TOE-2 in a fourth larval (L4) stage hermaphrodite. Closed arrowheads indicate mNeonGreen::TOE-2 localization to the apical surface of the germline cells. Open arrowheads indicate the positions of unknown cells near the vulva.
(TIF)

S3 Fig TOE-2 and NMY-2 are present at the apical germline. Confocal images of endogenously tagged TOE-2 and NMY-2 in a third larva stage (L3) hermaphrodite. Both proteins are expressed in the germline and accumulate at the apical surface of the germline cells. Arrowheads indicate the apical germline.
(TIF)

S4 Fig Intercellular bridges persist between Q lineage neuroblasts in *nmy-2(ts)* mutants. Anterior is to the left in A-C. A) QL.a and QL.p cells in an *nmy-2(ne3409ts)* mutant raised at the nonpermissive temperature with persistent intercellular bridges between their daughter cells. The cells on the left are the QL.p daughters. The QL.a daughters are more posterior because QL.a migrated past the Q.p cell before dividing. B) QL.a cell in an *nmy-2(ne3409ts)* mutant raised at the nonpermissive temperature with a persistent intercellular bridge between its daughter cells. The cell to the left is an undivided QL.p cell. C) QR.p cell in *nmy-2(ne1490ts)* mutant raised at the nonpermissive temperature with a persistent intercellular bridge between its daughter cells. Arrowheads indicate intercellular bridges, * indicates the QL.p and ^ indicates the QL.a cell.
(TIF)

S1 Table Strains used in this study. All strains used in this study with strain names, genotypes, and the figures that show data from these strains.
(XLSX)

S2 Table Comparison of posterior vs anterior intensity during Q lineage cell divisions. Comparison between Posterior and Anterior protein distributions. N is the number of cells measured in each phase for that genotype and cell type included in the line scan analysis. The average P/A Ratio is derived by averaging the ratio of the average of posterior and average of anterior normalized intensity ratios of each cell at each phase included in the line scan analysis. Empairs P/A estimate is derived from pairwise contrasts between the posterior and anterior effect sizes at each phase for each GLMM. Asymmetry indicates whether the posterior or anterior had significantly higher normalized intensity ratios with significance derived from the empairs analysis. (* p<0.05, ** p<0.01, *** p<0.001).
(XLSX)

S3 Table Coefficient estimates for models of the distribution of NMY-2::GFP, PIG-1::mNeonGreen, and mNeonGreen::TOE-2 during the Q lineage divisions. Table showing the estimated coefficients and confidence intervals for each variable in the models run for Q.a and Q.p for each genotype.
(XLSX)

S4 Table Mitotic potential in *pig-1*, *toe-2*, and temperature sensitive *nmy-2* mutants. Cell counts with extra QL.pp daughter cells were divided between those in which the QL.pp daughter survived but did not divide, resulting in either 2 SDQLs or 2 PVMs and those where the QL.pp daughter survived and divided, resulting in 2 of both cell types. This determination of QL.pp mitotic potential makes the assumption that when there is an extra SDQL or PVM, the extra cell results from a QL.pp surviving and adopting the SDQL or PVM fate and that when there is both an extra SDQL and a PVM, the extra cells result from a QL.pp surviving and dividing to produce an SDQL and PVM.
(XLSX)

## References

[1] Horvitz HR, Herskowitz I. Mechanisms of asymmetric cell division: Two Bs or not two Bs, that is the question. Cell. 1992;68(2):237–55. doi: 10.1016/0092-8674(92)90468-r 1733500

[2] Teuliere J, Garriga G. The Caenorhabditis elegans HAM-1 protein modifies G protein signaling and membrane extension to reverse the polarity of asymmetric cell division. bioRxiv. 2018;

[3] Ou G, Stuurman N, D’Ambrosio M, Vale RD. Polarized myosin produces unequal-size daughters during asymmetric cell division. Science (80-) [Internet]. 2010;330(6004):677–80. Available from: papers3://publication/doi/10.1126/science.1196112 20929735 10.1126/science.1196112 PMC3032534

[4] Feng G, Yi P, Yang Y, Chai Y, Tian D, Zhu Z, et al. Developmental stage-dependent transcriptional regulatory pathways control neuroblast lineage progression. Dev. 2013;140(18):3838–47. doi: 10.1242/dev.098723 23946438

[5] Chien SC, Brinkmann EM, Teuliere J, Garriga G. Caenorhabditis elegans PIG-1/MELK acts in a conserved PAR-4/LKB1 polarity pathway to promote asymmetric neuroblast divisions. Genetics. 2013;193(3):897–909. doi: 10.1534/genetics.112.148106 23267054 PMC3584005

[6] Gurling M, Talavera K, Garriga G. The DEP domain-containing protein TOE-2 promotes apoptosis in the Q lineage of C. Elegans through two distinct mechanisms. Dev [Internet]. 2014;141(13):2724–34. Available from: http://dev.biologists.org/cgi/doi/10.1242/dev.110486 10.1242/dev.110486 PMC4067965 24961802

[7] Ganguly R, Mohyeldin A, Thiel J, Kornblum HI, Beullens M, Nakano I. MELK—a conserved kinase: functions, signaling, cancer, and controversy. Clin Transl Med. 2015;4(1). doi: 10.1186/s40169-014-0045-y 25852826 PMC4385133

[8] Pacquelet A, Uhart P, Tassan JP, Michaux G. PAR-4 and anillin regulate myosin to coordinate spindle and furrow position during asymmetric division. J Cell Biol. 2015;210(7):1085–99. doi: 10.1083/jcb.201503006 26416962 PMC4586735

[9] Cordes S, Frank CA, Garriga G. The C. elegans MELK ortholog PIG-1 regulates cell size asymmetry and daughter cell fate in asymmetric neuroblast divisions. Development. 2006;133(14):2747–56. doi: 10.1242/dev.02447 16774992

[10] Wei H, Lambie EJ, Osório DS, Carvalho AX, Conradt B. PIG-1 MELK-dependent phosphorylation of nonmuscle myosin II promotes apoptosis through CES-1 Snail partitioning. PLoS Genet. 2020;16(9):1–27. doi: 10.1371/journal.pgen.1008912 32946434 PMC7527206

[11] Denning DP, Hatch V, Robert Horvitz H. Programmed elimination of cells by caspase-independent cell extrusion in C. elegans. Nature. 2012;488(7410):226–30. doi: 10.1038/nature11240 22801495 PMC3416925

[12] Arur S, Ohmachi M, Nayak S, Hayes M, Miranda A, Hay A, et al. Multiple ERK substrates execute single biological processes in Caenorhabditis elegans germ-line development. Proc Natl Acad Sci U S A. 2009;106(12):4776–81. doi: 10.1073/pnas.0812285106 19264959 PMC2660749

[13] Consonni S V, Maurice MM, Bos JL. DEP domains: structurally similar but functionally different. Nat Rev Mol Cell Biol [Internet]. 2014;15(5):357–62. Available from: doi: 10.1038/nrm3791 24739740

[14] Gibrat JF, Madej T, Bryant SH. Surprising similarities in structure comparison. Curr Opin Struct Biol. 1996;6(3):377–85. doi: 10.1016/s0959-440x(96)80058-3 8804824

[15] Jumper J, Evans R, Pritzel A, Green T, Figurnov M, Ronneberger O, et al. Highly accurate protein structure prediction with AlphaFold. Nature [Internet]. 2021;596(7873):583–9. Available from: doi: 10.1038/s41586-021-03819-2 34265844 PMC8371605

[16] Varadi M, Anyango S, Deshpande M, Nair S, Natassia C, Yordanova G, et al. AlphaFold Protein Structure Database: Massively expanding the structural coverage of protein-sequence space with high-accuracy models. Nucleic Acids Res. 2022;50(D1):D439–44. doi: 10.1093/nar/gkab1061 34791371 PMC8728224

[17] Liro MJ, Rose LS. Mitotic spindle positioning in the EMS cell of Caenorhabditis elegans requires LET-99 and LIN-5/NuMA. Genetics. 2016;204(3):1177–89. doi: 10.1534/genetics.116.192831 27672093 PMC5105850

[18] Tsou MFB, Hayashi A, DeBella LR, McGrath G, Rose LS. LET-99 determines spindle position and is asymmetrically enriched in response to PAR polarity cues in C. elegans embryos. Development. 2002;129(19):4469–81. doi: 10.1242/dev.129.19.4469 12223405

[19] Guo S, Kemphues KJ. A non-muscle myosin required for embryonic polarity in Caenorhabditis elegans. Nature. 1996;382(6590):455–8. doi: 10.1038/382455a0 8684486

[20] Tsankova A, Pham TT, Garcia DS, Otte F, Cabernard C. Cell Polarity Regulates Biased Myosin Activity and Dynamics during Asymmetric Cell Division via Drosophila Rho Kinase and Protein Kinase N. Dev Cell [Internet]. 2017;42(2):143–155.e5. Available from: doi: 10.1016/j.devcel.2017.06.012 28712722

[21] Cabernard C, Prehoda KE, Doe CQ. A spindle-independent cleavage furrow positioning pathway. Nature. 2010;467(7311):91–4. doi: 10.1038/nature09334 20811457 PMC4028831

[22] Pacquelet A. Asymmetric cell division in the one-cell C. Elegans embryo: Multiple steps to generate cell size asymmetry. In: Results and Problems in Cell Differentiation. 2017. p. 115–40. doi: 10.1007/978-3-319-53150-2_5 28409302

[23] Brenner S. The Genetics of Caenorhabditis elegans. Genetics [Internet]. 1974 May 1;77(1):71–94. Available from: https://academic.oup.com/genetics/article/77/1/71/5991065 doi: 10.1093/genetics/77.1.71 4366476 PMC1213120

[24] Liu J, Maduzia LL, Shirayama M, Mello CC. NMY-2 maintains cellular asymmetry and cell boundaries, and promotes a SRC-dependent asymmetric cell division. Dev Biol [Internet]. 2010;339(2):366–73. Available from: doi: 10.1016/j.ydbio.2009.12.041 20059995 PMC2903000

[25] Dickinson DJ, Ward JD, Reiner DJ, Goldstein B. Engineering the Caenorhabditis elegans genome using Cas9-triggered homologous recombination. Nat Methods. 2013;10(10):1028–34. doi: 10.1038/nmeth.2641 23995389 PMC3905680

[26] Zhu Z, Liu J, Yi P, Tian D, Chai Y, Li W, et al. A proneural gene controls C. elegans neuroblast asymmetric division and migration. FEBS Lett. 2014;588(7):1136–43. doi: 10.1016/j.febslet.2014.02.036 24589937

[27] Schindelin J, Arganda-Carreras I, Frise E, Kaynig V, Longair M, Pietzsch T, et al. Fiji: An open-source platform for biological-image analysis. Nat Methods. 2012;9(7):676–82. doi: 10.1038/nmeth.2019 22743772 PMC3855844

[28] Hadfield JD. MCMCglmm: MCMC Methods for Multi-Response GLMMs in R. J Stat Softw [Internet]. 2010;33(2):1–22. Available from: http://www.jstatsoft.org/20808728

[29] Dean CB, Nielsen JD. Generalized linear mixed models: A review and some extensions. Lifetime Data Anal. 2007 Dec;13(4):497–512. doi: 10.1007/s10985-007-9065-x 18000755

[30] Bolker BM, Brooks ME, Clark CJ, Geange SW, Poulsen JR, Stevens MHH, et al. Generalized linear mixed models: a practical guide for ecology and evolution. Trends Ecol Evol [Internet]. 2009 Mar;24(3):127–35. Available from: https://linkinghub.elsevier.com/retrieve/pii/S0169534709000196 doi: 10.1016/j.tree.2008.10.008 19185386

[31] Lenth R. Emmeans: estimated marginal means [Internet]. R package version 1.4.2. 2019. p. https://cran.r-project.org/package=emmeans. Available from: https://github.com/rvlenth/emmeans%0Ahttps://cran.r-project.org/package=emmeans

[32] Searle SR, Speed FM, Milliken GA. Population Marginal Means in the Linear Model: An Alternative to Least Squares Means. Am Stat [Internet]. 1980 Nov 30;34(4):216–21. Available from: http://www.tandfonline.com/doi/abs/10.1080/00031305.1980.10483031

[33] Wickham H. Tidy Data. J Stat Softw [Internet]. 2014;59(10):1–23. Available from: http://www.jstatsoft.org/26917999

[34] Wickham H. ggplot2: Elegant Graphics for Data Analysis [Internet]. Vol. 35, Journal of Statistical Software. Springer-Verlag New York; 2010. Available from: https://ggplot2.tidyverse.org

[35] Sulston JE, Horvitz HR. Post-embryonic cell lineages of the nematode, Caenorhabditis elegans. Dev Biol. 1977;56(1):110–56. doi: 10.1016/0012-1606(77)90158-0 838129

[36] Coffman VC, Kachur TM, Pilgrim DB, Dawes AT. Antagonistic Behaviors of NMY-1 and NMY-2 Maintain Ring Channels in the C. elegans Gonad. Biophys J [Internet]. 2016;111(10):2202–13. Available from: 10.1016/j.bpj.2016.10.011 27851943 PMC5113261

[37] Wernike D, Chen Y, Mastronardi K, Makil N, Piekny A. Mechanical forces drive neuroblast morphogenesis and are required for epidermal closure. Dev Biol [Internet]. 2016;412(2):261–77. Available from: doi: 10.1016/j.ydbio.2016.02.023 26923492

[38] Mishra N, Wei H, Conradt B. Caenorhabditis elegans ced-3 caspase is required for asymmetric divisions that generate cells programmed to die. Genetics. 2018;210(3):983–98. doi: 10.1534/genetics.118.301500 30194072 PMC6218217

[39] Deng Q, Wang H. Re-visiting the principles of apicobasal polarity in Drosophila neural stem cells. Dev Biol [Internet]. 2022;484(November 2021):57–62. Available from: doi: 10.1016/j.ydbio.2022.02.006 35181298

[40] LaFoya B, Prehoda KE. Actin-dependent membrane polarization reveals the mechanical nature of the neuroblast polarity cycle. Cell Rep [Internet]. 2021;35(7):109146. Available from: doi: 10.1016/j.celrep.2021.109146 34010656 PMC8174105

[41] Barros CS, Phelps CB, Brand AH. Drosophila nonmuscle myosin II promotes the asymmetric segregation of cell fate determinants by cortical exclusion rather than active transport. Dev Cell. 2003;5(6):829–40. doi: 10.1016/s1534-5807(03)00359-9 14667406

