# Supplementary figures and images for: NMY-2, TOE-2 and PIG-1 regulate Caenorhabditis elegans asymmetric cell divisions
Source: PLoS One. 2024 May 24;19(5):e0304064. doi: 10.1371/journal.pone.0304064 (PMC11125515; doi:10.1371/journal.pone.0304064)

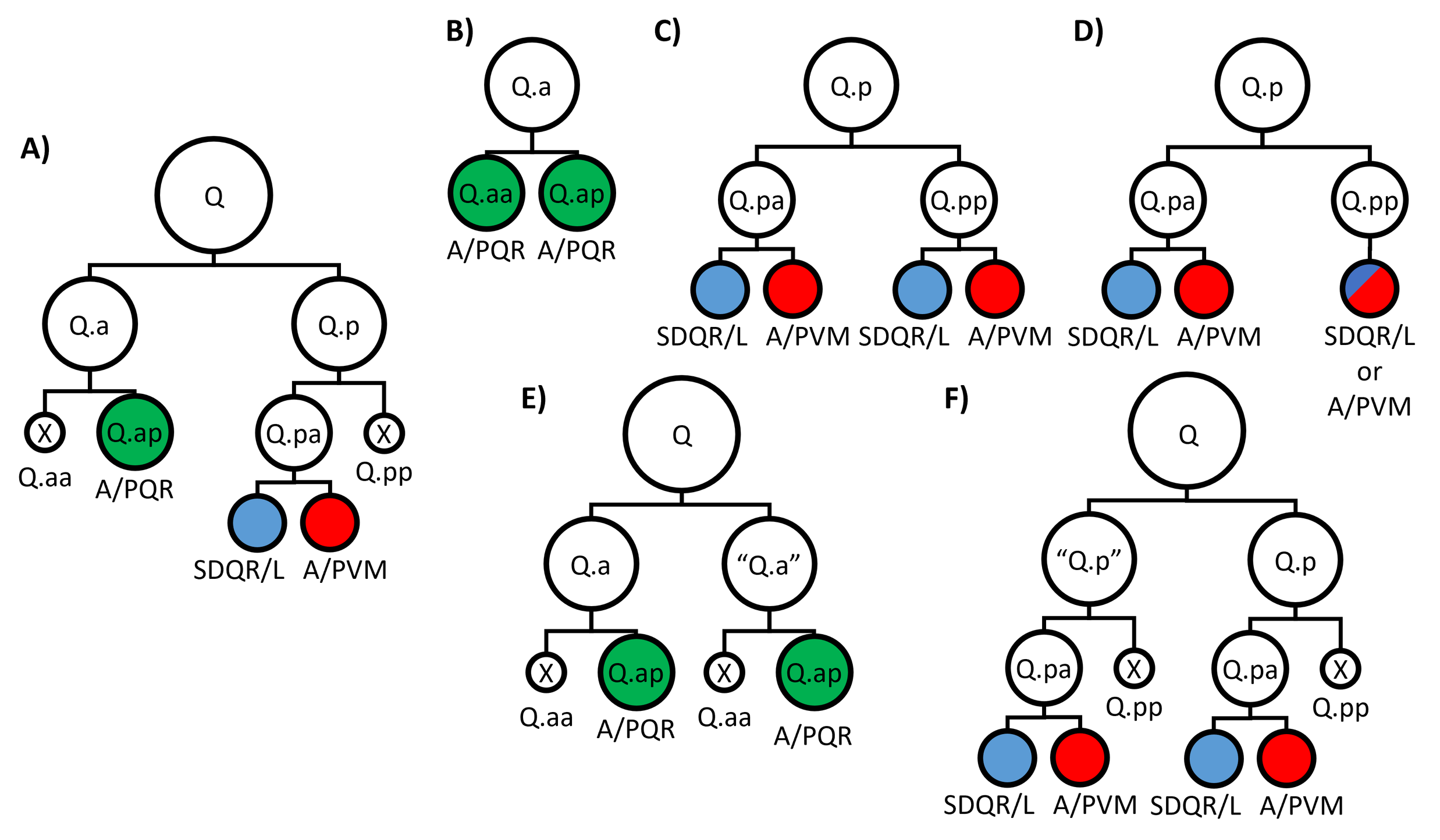

Supplement: S1 Fig — A) The wild-type Q lineage. B) A Q.a division defect where the Q.aa fails to die and adopts the Q.ap fate as an A/PQR neuron. C) A Q.p division defect where the Q.pp fails to die and adopts the Q.pa fate. D) A Q.p division defect where the Q.pp fails to die but does not divide and adopts an SDQR/L or A/PVM fate. E) A Q division defect where Q.p adopts the fate of Q.a. This results in an extra A/PQR and an absence of the Q.p descendants. F) A Q division defect, where the Q.a adopts the fate of Q.p. This results in a duplication of the SDQR/L and A/PVM neurons and an absence of the A/PQR. The Q.p transformation in E and the Q.a transformation in F may also display the defects shown in B, and C and D respectively, resulting in three or more neurons that express a specific fate. (TIF) [file pone.0304064.s001.tif]

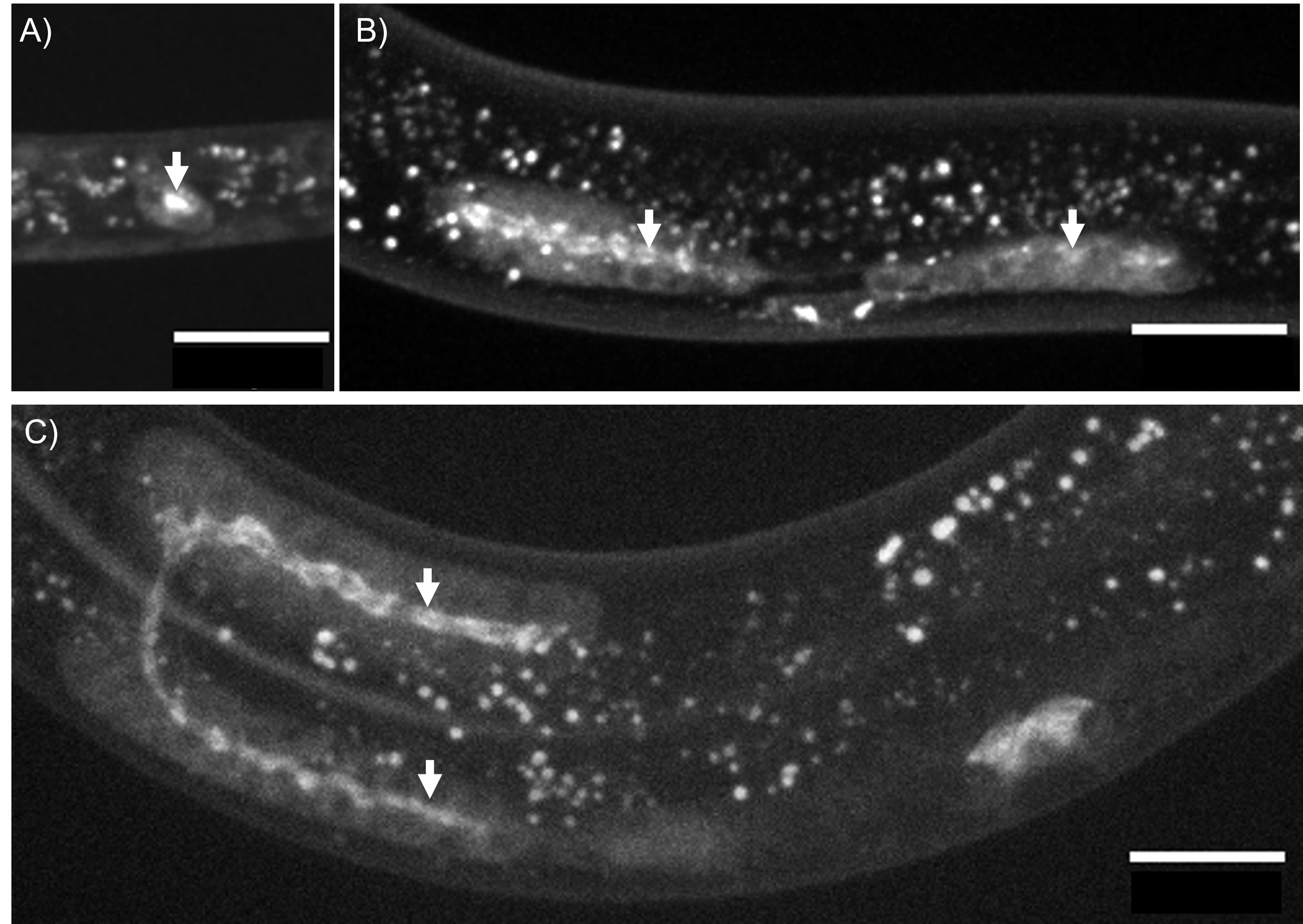

Supplement: S2 Fig — A) mNeonGreen::TOE-2 in a first larval (L1) stage hermaphrodite. Closed arrowhead indicates mNeonGreen::TOE-2 at the point of contact between Z2 and Z3. B) mNeonGreen::TOE-2 in an L3 stage hermaphrodite. Closed arrowheads indicate localization of mNeonGreen::TOE-2 to the apical surface of the germline cells. Open arrowheads indicate mNeonGreen::TOE-2 localization to unknown cells near the vulva. C) mNeonGreen::TOE-2 in a fourth larval (L4) stage hermaphrodite. Closed arrowheads indicate mNeonGreen::TOE-2 localization to the apical surface of the germline cells. Open arrowheads indicate the positions of unknown cells near the vulva. (TIF) [file pone.0304064.s002.tif]

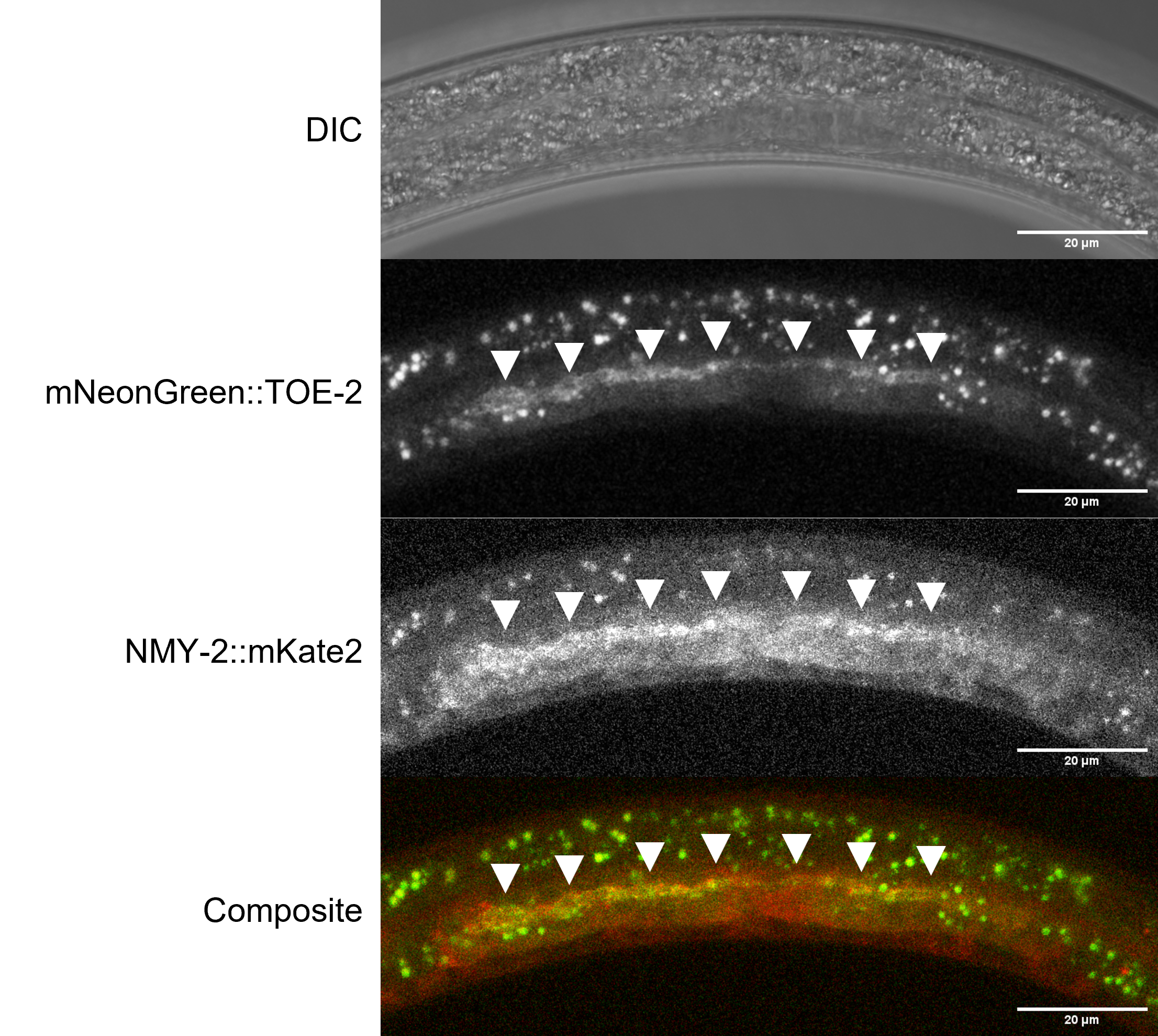

Supplement: S3 Fig — Confocal images of endogenously tagged TOE-2 and NMY-2 in a third larva stage (L3) hermaphrodite. Both proteins are expressed in the germline and accumulate at the apical surface of the germline cells. Arrowheads indicate the apical germline. (TIF) [file pone.0304064.s003.tif]

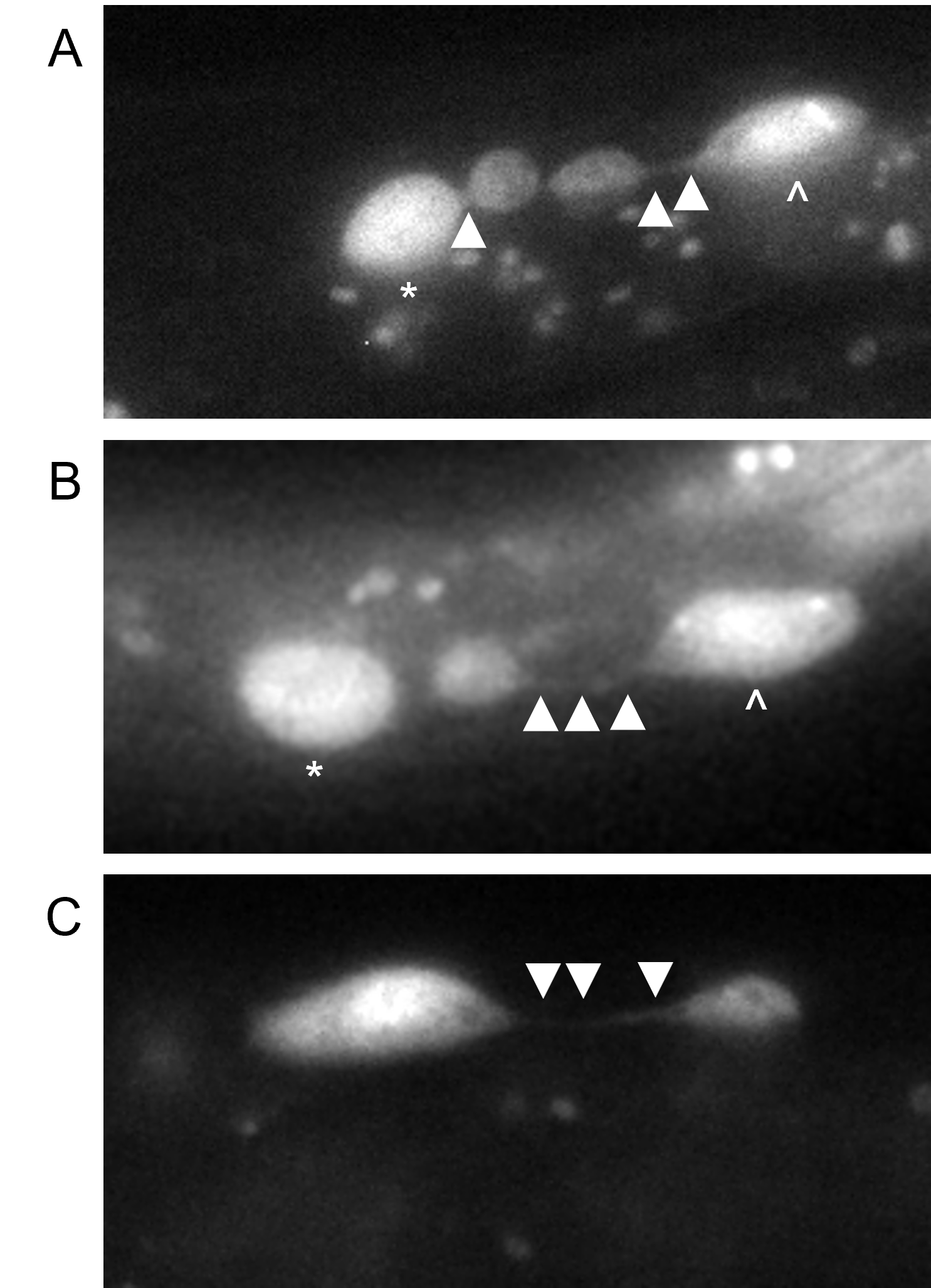

Supplement: S4 Fig — Anterior is to the left in A-C. A) QL.a and QL.p cells in an nmy-2(ne3409ts) mutant raised at the nonpermissive temperature with persistent intercellular bridges between their daughter cells. The cells on the left are the QL.p daughters. The QL.a daughters are more posterior because QL.a migrated past the Q.p cell before dividing. B) QL.a cell in an nmy-2(ne3409ts) mutant raised at the nonpermissive temperature with a persistent intercellular bridge between its daughter cells. The cell to the left is an undivided QL.p cell. C) QR.p cell in nmy-2(ne1490ts) mutant raised at the nonpermissive temperature with a persistent intercellular bridge between its daughter cells. Arrowheads indicate intercellular bridges, * indicates the QL.p and ^ indicates the QL.a cell. (TIF) [file pone.0304064.s004.tif]
